# MARCH8-mediated ubiquitination regulates expression of the antiviral protein IFITM3

**DOI:** 10.1016/j.jbc.2025.110879

**Published:** 2025-11-04

**Authors:** Liang Wei, Fei Zhao, Xiaoman Liu, Shan Mei, Yu Huang, Yu Xie, Yamei Hu, Liming Wang, Lingwa Wang, Zhao Gao, Chen Chen, Yueyue Shi, Yurong He, Jiaxun Wang, Tiffany Xue, Fengwen Xu, Jugao Fang, Fei Guo

**Affiliations:** 1 Key Laboratory of Pathogen Infection Prevention and Control (Ministry of Education), State Key Laboratory of Respiratory Health and Multimorbidity, National Institute of Pathogen Biology, Chinese Academy of Medical Sciences & Peking Union Medical College, Beijing, PR China; 2 NHC Key Laboratory of Systems Biology of Pathogens, National Institute of Pathogen Biology and Center for AIDS Research, Chinese Academy of Medical Sciences & Peking Union Medical College, Beijing, PR China; 3 Department of Otolaryngology Head and Neck Surgery, Beijing Tongren Hospital, Capital Medical University, Beijing, PR China; 4 Department of Chemistry, Bryn Mawr College, Bryn Mawr, Pennsylvania, USA

**Keywords:** MARCH8, E3 ligase, IFITM3, ubiquitination, virus infection, IFN

## Abstract

The Membrane-Associated RING-CH (MARCH) family of E3 ubiquitin ligases modulates membrane protein stability and contributes to host antiviral immune responses. Their functions largely depend on the nature of protein substrates. In this study, we identified interferon-induced transmembrane protein 3 (IFITM3), a critical antiviral effector protein, as a novel substrate of MARCH8 by coimmunoprecipitation coupled with LC–MS/MS analysis. Mechanistically, MARCH8 promotes the lysosomal degradation of IFITM3 primarily through K63-linked polyubiquitination at lysine 24, which facilitates its trafficking and turnover from the plasma membrane to endosomes and lysosomes. MARCH8-KO cells exhibit plasma membrane accumulation of IFITM3 compared with WT controls after interferon treatment. Functionally, MARCH8 expression attenuated the IFITM3-mediated restriction of vesicular stomatitis virus and influenza A virus entry, thereby increasing cell susceptibility to viral infection. Together, these findings establish a novel regulatory mechanism whereby MARCH8 modulates innate immunity through regulating the trafficking and turnover of antiviral protein IFITM3.

The Membrane-Associated RING-CH (MARCH) family of E3 ubiquitin (Ub) ligases are characterized by a conserved N-terminal RING-CH (C4HC3) motif. They have been reported to regulate the properties of many cellular membrane proteins, including their stability, trafficking, and functions (1, 2, 3, 4, 5). MARCH8 was initially identified as cellular modulators of immune recognitions (MIRs) because of its structural and functional homology with the MIR-1 (K3) and MIR-2 (K5) proteins of Kaposi’s sarcoma–associated herpesvirus; they are all membrane-associated E3 ligases (6, 7, 8, 9).

A group of diverse cellular membrane proteins are subject to the regulation of MARCH8 through ubiquitination. The first substrate of MARCH8 was reported to be the major histocompatibility complex-II. The Lys225 amino acid in the cytoplasmic tail of major histocompatibility complex-II is ubiquitinated by MARCH8, leading to recognition by the endosomal sorting complexes required for transport and subsequent degradation in lysosomes (1, 10). More proteins have been identified as substrates of MARCH8, such as CD86, CD98, CD81, CD44, and transferrin receptor (2, 4, 8, 11, 12). MARCH8 has been further shown to interact with tumor necrosis factor–related apoptosis-inducing ligand receptor 1 (TRAIL-R1/DR4), ubiquitinate the Lys273 amino acid, and modulate apoptosis signaling (5, 13). MARCH8 also interacts with programmed death ligand 1 through its N-terminal region, ubiquitinates and degrades programmed death ligand 1, thereby regulating immune responses (14). Moreover, Chen *et al.* (3) reported that MARCH8 interacts with interleukin-1 receptor accessory protein (IL1RAP), catalyzes K48-linked polyubiquitination at Lys512 and subsequent degradation, which in turn regulates interleukin-1β–induced NF-κB and mitogen-activated protein kinase signaling pathways.

MARCH8 also targets viral membrane proteins, thus playing a significant role in antiviral defense. For example, MARCH8 downregulates HIV-1 envelope (Env) glycoprotein (GP), preventing Env incorporation into virions, an antiviral activity that is shared by MARCH1 and MARCH2 (15, 16, 17, 18). Similarly, MARCH8 causes ubiquitination and degradation of vesicular stomatitis virus (VSV) GP, murine leukemia virus Env GP, spring viremia of carp virus G-GP, and raby virus GP, which leads to impairment in virion infectivity (15, 16, 19, 20, 21, 22, 23). We previously reported that MARCH8 targets influenza A virus (IAV) matrix 2 (M2) protein for lysosomal degradation, thereby preventing virion release from the infected cells (24). Mechanistically, MARCH8 also impedes the cleavage and maturation of HIV-1 Env GP, Ebola virus (EBOV) GP, IAV H5N1 HA, and severe acute respiratory syndrome coronavirus 2 by interfering with cellular protease furin (19, 21, 22, 25). Furthermore, MARCH8’s impact extends to the HA–M1–M2 complex in IAV H1N1 virions, impeding virus assembly (26). Instead of being inhibited by MARCH8, some viruses, such as hepatitis C virus (HCV), dengue virus, and Zika virus, hijack MARCH8 for viral protein ubiquitination to facilitate replication (27, 28). These complex roles of MARCH8 in host antiviral defense are further supported by the finding that MARCH8 is able to suppress interferon (IFN) production through targeting cGAS (29).

To gain more insights into the multifaceted functions of MARCH8 in regulating key cellular processes, including antiviral defense, we employed proteomics to identify cellular proteins that are potentially associated with MARCH8. The antiviral protein IFITM3 was found to interact with MARCH8 and further ubiquitinated and degraded *via* the lysosomal pathway. MARCH8-mediated ubiquitination of IFITM3 occurs primarily at lysine 24 (Lys24; K24) *via* the K63 linkage and is crucial for the trafficking and turnover of IFITM3 from the plasma membrane to endosomes and lysosomes. Functionally, MARCH8 KO increases IFITM3 levels, thus rendering cells more resistant to the infection of VSV and IAV.

## Results

### Identification of IFITM3 as a substrate of MARCH8

**Figure 1:**
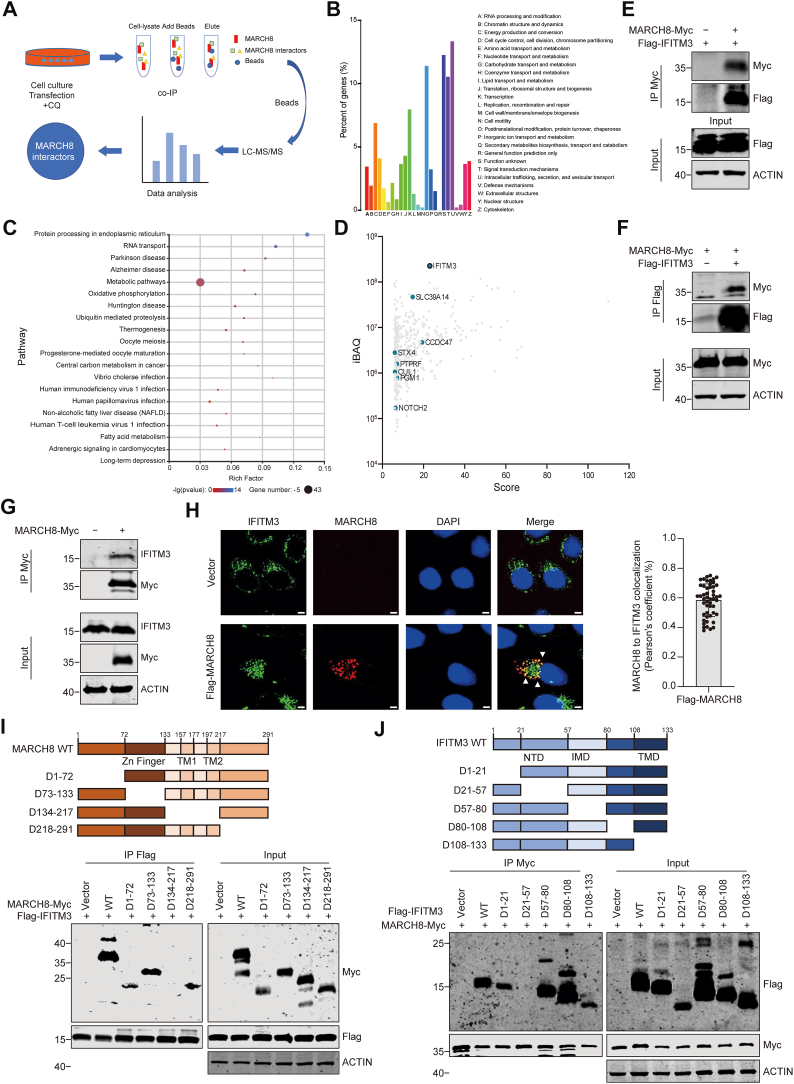
**Identification of IFITM3 as an interactor of MARCH8.***A,* schematic representation of the identification process. HeLa cells were transfected with MARCH8 plasmid or empty vector. Cells were then treated with lysosomal inhibitor chloroquine (CQ). Potential interactors of MARCH8 were identified by LC–MS/MS analysis of MARCH8 co-IP protein samples. The empty vector group was used as a negative control. *B,* KEGG pathway bubble diagram of top 20 differentially expressed proteins. The rich factor represents the enrichment factor, which is the ratio of the proportion of differentially expressed proteins annotated to the pathway to the proportion of background proteins annotated to the pathway. *C,* COG function classification of unigenes shows 25 different functional groups. *D,* differential proteins were nalysed according to mass spectrum score (score) and iBAQ protein intensity. Proteins known to interact with MARCH8 are highlighted in *light blue*. *E* and *F,* reciprocal co-IP analysis showed the interaction between MARCH8 and IFITM3 in HEK293T cells. *G,* co-IP analysis showed the interaction between MARCH8 and endogenous IFITM3 in HeLa cells. *H,* HeLa cells were transfected with FLAG-MARCH8 or empty vector plasmid. Cells were treated with CQ (50 μM) for 4 h and processed for immunofluorescence staining with anti-IFITM3 and anti-FLAG antibodies. Scale bars indicate 5 μm in all panels. Statistical result of the colocalization analysis is shown on the *right*. Values are means ± SD from >50 cells (n = 3 independent experiments). Statistical differences were determined by two-sided Student’s *t* test. *I* and *J,* co-IP and Western blotting analysis of HEK293T cells were performed after transfection with plasmids encoding FLAG-IFITM3 and full-length MARCH8-Myc or its truncation mutants (*I*) or with plasmids encoding MARCH8-Myc and full-length FLAG-IFITM3 or its truncation mutants (*J*). Schematic diagrams of MARCH8 (*I*) and IFITM3 (*J*) and their truncation mutants are shown above the Western blots. COG, clusters of orthologous groups of proteins; co-IP, coimmunoprecipitation; HEK293T, human embryonic kidney 293T cell line; iBAQ, intensity-based absolute quantification; IFITM3, interferon-induced transmembrane protein 3; KEGG, Kyoto Encyclopedia of Genes and Genomes; MARCH, Membrane-Associated RING-CH.

To identify potential target proteins of MARCH8, we immunoprecipitated (IP) MARCH8 from cells that were treated with the lysosomal inhibitor, chloroquine (CQ), considering that MARCH8 is known to degrade proteins through the lysosomal pathway. Results of LC–MS/MS revealed 421 differential proteins that are potentially associated with MARCH8 compared with the negative control (Fig. 1*A*). Bioinformatics analysis showed that these 421 proteins were enriched in protein processing, RNA transport, Alzheimer’s disease, Parkinson’s disease, and other signaling pathways (Fig. 1*B*). The functions of these proteins were mainly enriched in intracellular transport, sorting, vesicle transport, signal transduction, post-translational modification, protein turnover, molecular chaperones, transcription, and energy production and conversion (Figs. 1*C* and S1). Some of the previously reported MARCH8-interacting proteins, including CUL1, STX4, CCDC47, NOTCH2, PGM1, PTPRF, and SLC39A14, were among these 421 proteins (Table S1) (30, 31, 32). By analyzing mass spectrometry (MS) parameters, including confidence score and protein abundance (intensity-based absolute quantification), top-ranking hits include IFITM3, RCN1, RLA2, and BAG3 (Fig. 1*D*). IFITM3, a well-studied IFN-induced antiviral protein, localizes to endosomes, lysosomes, and the plasma membrane (33, 34). It plays a crucial role in various physiological and pathological processes, including tumorigenesis, immune signaling, B-cell activation, development, and Alzheimer’s disease progression, and was thus selected for further study (35, 36, 37, 38, 39, 40, 41, 42, 43, 44).

To confirm the interaction between IFITM3 and MARCH8, we performed co-IP experiments in human embryonic kidney 293T (HEK293T) cells expressing both proteins. The results showed reciprocal coprecipitation of MARCH8 and IFITM3 in cotransfected cells, and MARCH8 also interacted with endogenous IFITM3 in HeLa cells (Fig. 1, *E*–*G*). The co-IP data are corroborated by the colocalization of MARCH8 and IFITM3 in HeLa cells (Fig. 1*H*). In addition, the direct interaction between MARCH8 and IFITM3 was confirmed by bimolecular fluorescence complementation (BiFC) assay (45) (Fig. S2). MARCH8 has a conserved RING-CH domain and two transmembrane domains (TMDs). To investigate the role of these domains in the interaction with IFITM3, we generated four truncation mutants: an N-terminal deletion, a zinc finger deletion, a TMD deletion, and a C-terminal deletion containing both the zinc finger and TMDs. Co-IP data showed that the TMD deletion led to the loss of the interaction with IFITM3, indicating that the TMDs are crucial for MARCH8’s binding to IFITM3 (Fig. 1*I*). Human IFITM3 has a long amino-terminal domain (N-terminal domain), an intramembrane domain, a short intracellular conserved loop (CIL), a TMD, and a C-terminal domain. We constructed five truncation mutants that selectively lack sequences in these domains. The mutant lacking the N-terminal amino acids 21 to 57 lost interaction with MARCH8 (Fig. 1*J*). Collectively, these findings confirmed that MARCH8 directly interacts with IFITM3.

### MARCH8 leads to IFITM3 degradation in lysosomes through the K63-linked polyubiquitylation

**Figure 2:**
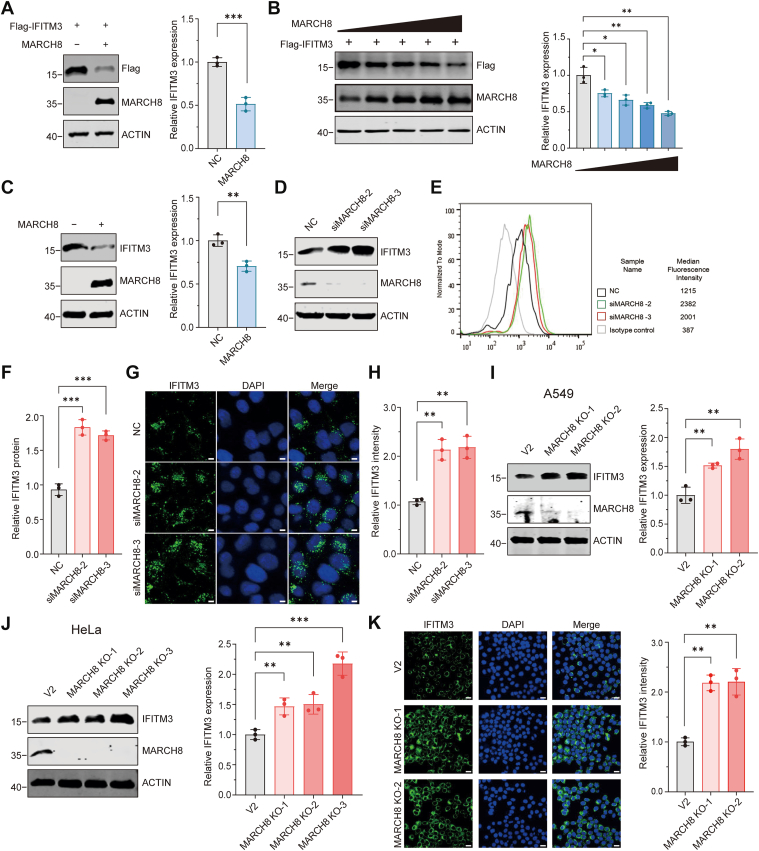
**Effects of MARCH8 on IFITM3 degradation.***A,*. HEK293T cells were cotransfected with FLAG-IFITM3 and WT MARCH8 (WT) or a control vector, followed by Western blot. Values are means ± SD from three independent experiments (n = 3). Statistical differences were determined by two-sided Student’s *t* test; ∗∗∗*p* < 0.0001. *B,* HEK293T cells were cotransfected with FLAG-IFITM3 and increasing amounts of MARCH8, followed by Western blot. Values are means ± SD from three independent experiments (n = 3). Statistical differences were determined by two-sided Student’s *t* test; ∗*p* < 0.01, ∗∗*p* < 0.001. *C,* HEK293T cells were transfected with WT MARCH8 (WT) or a control vector, followed by Western blot. Values are means ± SD from three independent experiments (n = 3). Statistical differences were determined by two-sided Student’s *t* test; ∗∗*p* < 0.001. *D*–*H,* HeLa cells were transfected with siRNA targeting MARCH8 or control siRNA. MARCH8 and IFITM3 expression was examined by Western blotting (*D*). IFITM3 expression was also examined by flow cytometry assay (*E*) and immunofluorescence (*G*). Scale bars indicate 20 μm in all panels. Values are means ± SD from three independent experiments (n = 3). Statistical differences were determined by two-sided Student’s *t* test; ∗∗∗*p* < 0.0001, ∗∗*p* < 0.001. *I*–*K,* the endogenous MARCH8 in A549 (*I*) and HeLa (*J*) cells was knocked out by lentiviral CRISPR–Cas9. MARCH8 and IFITM3 expression was examined by Western blotting (*I* and *J*). IFITM3 expression in HeLa cells was also examined by immunofluorescence (*K*). The quantification is shown on the *right*. Scale bars indicate 50 μm in all panels. Values are means ± SD from three independent experiments (n = 3). Statistical differences were determined by two-sided Student’s *t* test; ∗∗∗*p* < 0.0001, ∗∗*p* < 0.001. HEK293T, human embryonic kidney 293T cell line; IFITM3, interferon-induced transmembrane protein 3; MARCH, Membrane-Associated RING-CH.

To investigate the effect of MARCH8 on IFITM3 expression, we initially coexpressed IFITM3 and MARCH8 in HEK293T cells, which resulted in downregulation of IFITM3 levels (Fig. 2*A*). Subsequently, we coexpressed IFITM3 with increasing concentrations of MARCH8 in HEK293T cells, demonstrating that MARCH8 downregulates IFITM3 protein levels in a dose-dependent manner (Fig. 2*B*). To determine whether this effect extends to endogenous IFITM3, we overexpressed MARCH8 in HeLa cells and similarly observed a reduction in endogenous IFITM3 levels (Fig. 2*C*). Next, to assess the physiological role of endogenous MARCH8, we performed siRNA-mediated knockdown in both A549 and HeLa cells. Western blotting, flow cytometry, and immunofluorescence analyses demonstrated that depletion of MARCH8 increased endogenous IFITM3 expression in both cell lines (Fig. 2, *D*–*H*). To further confirm these findings, we generated stable MARCH8-KO cell lines in A549 and HeLa cells using CRISPR–Cas9 genome editing. Consistent with the knockdown results, genetic ablation of MARCH8 led to a significant increase in IFITM3 protein levels (Fig. 2, *I*–*K*).

**Figure 3:**
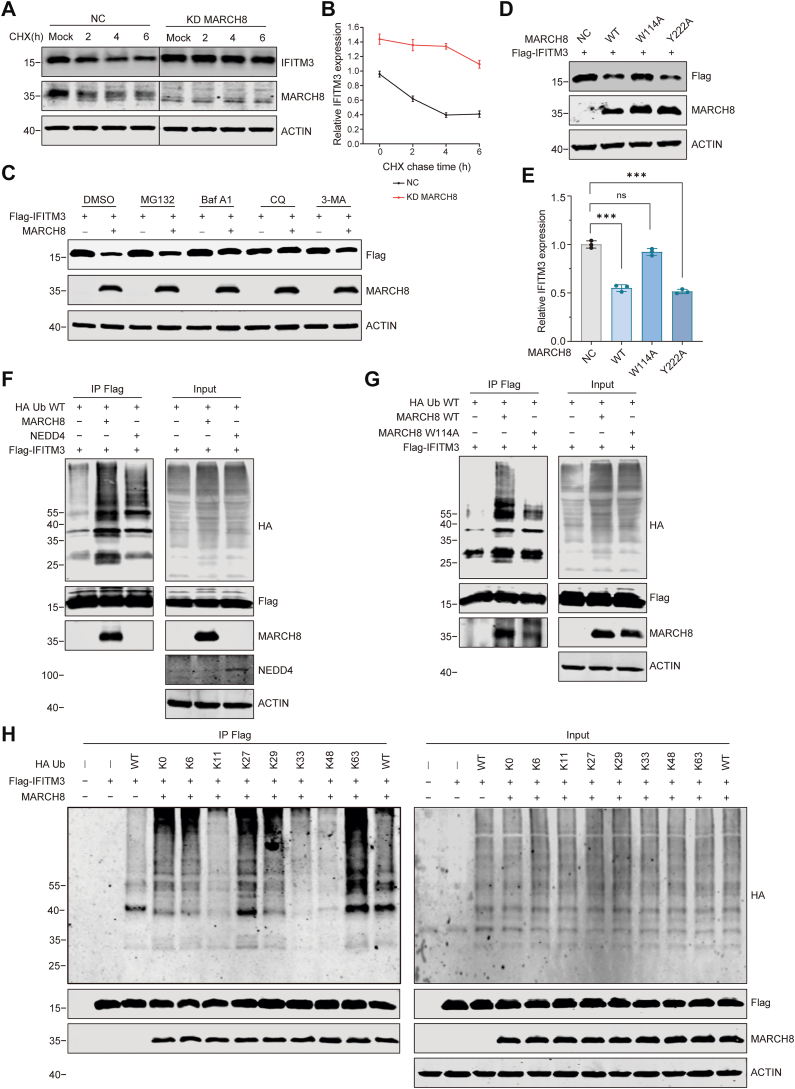
**MARCH8 leads to IFITM3 degradation in lysosomes through the K63-linked polyubiquitylation**. *A* and *B,* MARCH8 WT and knockdown (KD) HeLa cells were treated by CHX (100 μM) for 0, 2, 4, and 6 h followed by Western blot. Lanes shown are nonadjacent but from the same immunoblot. The quantification is shown on the *right* (*B*). Quantifications derive from three independent experiments (mean ± SD). *C,* HEK293T cells were cotransfected with FLAG-IFITM3 and MARCH8 WT or empty vector. Cells were then treated with DMSO, MG132 (50 μM), bafilomycin (1 μM), CQ (50 μM), and 3-MA (100 μM) for 4 h and processed for Western blot. *D,* HEK293T cells were cotransfected with FLAG-IFITM3 and WT MARCH8 (WT), the W114A mutant MARCH8 (W114A), or the Y222A mutant MARCH8 (Y222A) followed by Western blot. *E,* statistical results of the Western blot (*D*). Values are means ± SD from three independent experiments (n = 3). Statistical differences were determined by two-sided Student’s *t* test; ∗∗∗*p* < 0.0001. ns, nonsignificant. *F*, HEK293T cells were cotransfected for 24 h with HA-Ub, FLAG-IFITM3, and vector, MARCH8, or NEDD4. Cells were treated with CQ (50 μM) for 4 h. Cells were harvested for co-IP using anti-FLAG Ab-coated agarose beads and analyzed by Western blot. *G,* HEK293T cells were cotransfected for 24 h with HA-Ub, FLAG-IFITM3, and vector, MARCH8 WT, or MARCH8 W114A. Cells were treated with CQ (50 μM) for 4 h. Cells were harvested for co-IP using anti-FLAG beads and analyzed by Western blot. *H,* HEK293T cells were transfected with HA-tagged ubiquitin mutants, FLAG-IFITM3 and MARCH8. Cells were treated with CQ (50 μM) for 4 h. Cell lysates were subject to IP with anti-FLAG antibody, and the IP and input samples were analyzed by Western blot with antibodies against the indicated protein targets. Ab, antibody; CHX, cycloheximide; co-IP, coimmunoprecipitation; CQ, chloroquine; DMSO, dimethyl sulfoxide; HEK293T, human embryonic kidney 293T cell line; IFITM3, interferon-induced transmembrane protein 3; K63, lysine 63; MARCH, Membrane-Associated RING-CH; Ub, ubiquitin.

We then explored the mechanism by which MARCH8 downregulates IFITM3. We first measured the levels of IFITM3 mRNA in MARCH8 knockdown and KO cells, and no significant change was detected (Fig. S3). This suggests that MARCH8 downregulates IFITM3 expression at the protein level, not at the mRNA level. This conclusion was further supported by the data showing that knockdown of MARCH8 significantly alleviated the degradation rate of IFITM3 protein after treatment with the translation inhibitor cycloheximide (Fig. 3, *A* and *B*). Since MARCH8 targets its substrates to lysosomes for degradation, we tested whether MARCH8 also diminishes IFITM3 protein levels *via* this pathway. Indeed, coincubation of lysosomal inhibitors CQ (50 μM) and bafilomycin A1 (100 nM) alleviated the downregulation of IFITM3 by MARCH8, whereas the inhibitors for autophagy (3-MA) and proteasome (MG132) displayed a weak, not significant effect (Figs. 3*C*. S4, and S5). These findings suggest that MARCH8 regulates IFITM3 levels in a lysosome-dependent manner.

To investigate whether the downregulation of IFITM3 by MARCH8 is associated with its E3 ligase activity or endocytic activity, we constructed the E3 ligase–null mutant of MARCH8 (W114A) and the Y_222_XXL_224_ motif mutant of MARCH8 (Y222A), which is known to be recognized by the adaptor protein (AP) μ-subunits in the C-terminal CT domain and is involved in the antiviral activity of MARCH8 against HIV-1 Env (16, 46). IFITM3 degradation was lost for the W114A mutant but not affected by the Y222A mutant of MARCH8 (Fig. 3, *D* and *E*). And we also found that the localization of these two mutants was consistent with previous reports that W114A was mainly localized to the plasma membrane, whereas Y222A remained mainly intracellular (24, 47) (Fig. S6). These results suggest that MARCH8 reduces IFITM3 expression through its E3 ligase function. To test whether MARCH8 ubiquitinates IFITM3, we coexpressed MARCH8 and IFITM3 together with HA-Ub, which allows detection of ubiquitinated IFITM3 using anti-HA antibodies. We used CQ to slow down lysosomal degradation so that enough potentially ubiquitinated IFITM3 can be recovered for IP and Western blot analysis. The results showed a significant increase in IFITM3 ubiquitination when MARCH8 was overexpressed (Fig. 3*F*). NEDD4 has been reported to be able to ubiquitinate IFITM3 and was tested as a positive control (48). In addition, the W114A mutant significantly reduced the level of IFITM3 ubiquitination, further supporting that the ubiquitination level of IFITM3 was dependent on the E3 Ub ligase activity of MARCH8 (Fig. 3*G*). Together, these results demonstrate that MARCH8 ubiquitinates IFITM3 through its E3 ligase activity.

To determine which type of polyubiquitin linkage to IFITM3 is catalyzed by MARCH8, we transfected IFITM3 and MARCH8 into HEK293T cells together with each of the Ub mutants K6, K11, K27, K29, K33, K48, and K63, each of which contains only one lysine available for polyubiquitination. Co-IP and Western blot analyses showed that MARCH8-mediated K63-linked ubiquitination is the predominant modification of IFITM3, whereas K0-, K6-, K27-, and K29-linked ubiquitination exhibited weaker yet detectable signals (Fig. 3*H*). Collectively, these results demonstrate that MARCH8 primarily facilitates lysosomal degradation of IFITM3 *via* K63-linked polyubiquitination.

### K24 of IFITM3 is the primary ubiquitination site by MARCH8

**Figure 4:**
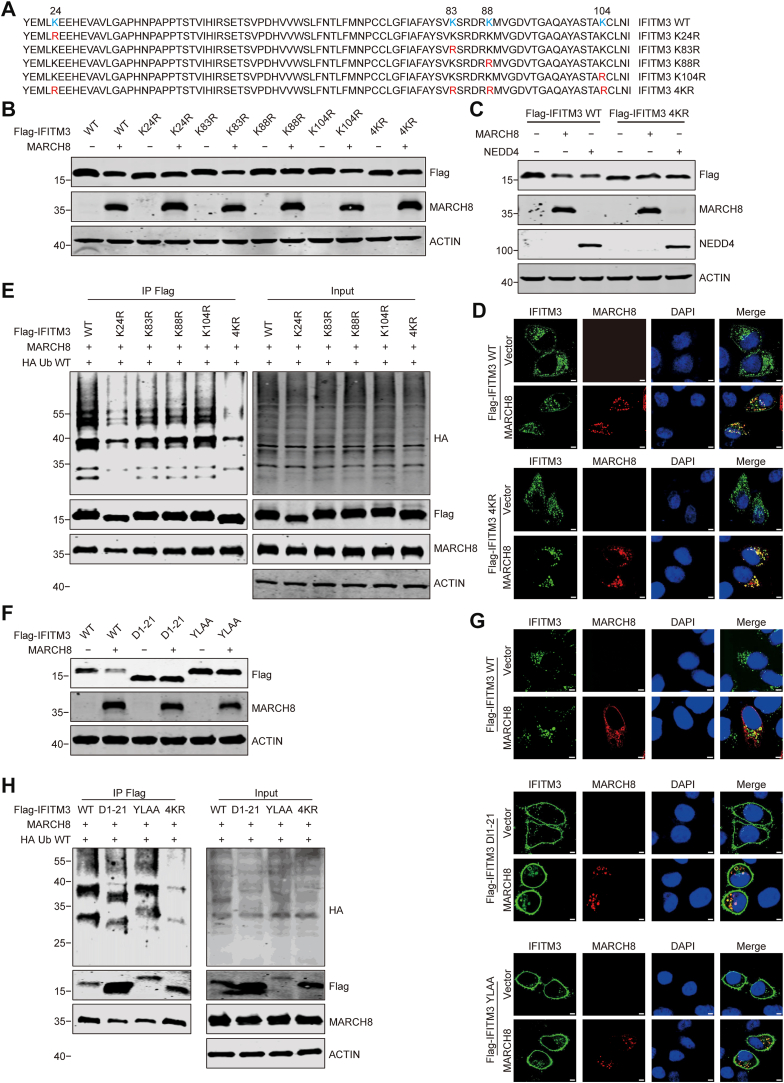
**K24 is the primary ubiquitination site of IFITM3 by MARCH8**. *A,* amino acid sequences of IFITM3 and the indicated mutants. Lysine residues (K)/arginine residue (R) mutants are marked as *red*. *B,* HEK293T cells were transfected with FLAG-IFITM3 mutants and vector or MARCH8 WT followed by Western blot. *C,* HEK293T cells were transfected with FLAG-IFITM3 WT or 4KR mutant and vector, MARCH8, or NEDD4 followed by Western blot. *D,* HeLa cells were cotransfected with FLAG-IFITM3 WT or 4KR mutant and vector or MARCH8. Cells were treated with CQ (50 μM) for 4 h and processed for immunofluorescence staining with anti-FLAG and anti-MARCH8 antibodies. Scale bars indicate 5 μm in all panels. *E,* ubiquitination of IFITM3 mutants by MARCH8. HEK293T cells were transfected with FLAG-IFITM3 mutants, HA Ub WT, and MARCH8. Cells were treated with CQ (50 μM) for 4 h. Cell lysates were subject to IP with anti-FLAG antibody, and the IP and input samples were analyzed by Western blotting with antibodies against the indicated protein targets. *F,* HEK293T cells were cotransfected with MARCH8 WT and FLAG-IFITM3 WT, FLAG-IFITM3 D1-21 mutants, or FLAG-IFITM3 YLAA mutants for Western blots. *G,* HeLa cells were cotransfected with MARCH8 WT and FLAG-IFITM3 WT, D1-21 mutants, or YLAA mutants. Cells were treated with CQ (50 μM) for 4 h and processed for immunofluorescence staining with anti-FLAG and anti-MARCH8 antibodies. Scale bars indicate 5 μm in all panels. *H,* HEK293T cells were transfected with FLAG-IFITM3 mutants D1-21 and YLAA, HA-Ub WT, and MARCH8. Cell lysates were subject to IP with anti-FLAG antibody, and the IP and input samples were analyzed by Western blotting with antibodies against the indicated protein targets. CQ, chloroquine; HEK293T, human embryonic kidney 293T cell line; IFITM3, interferon-induced transmembrane protein 3; IP, immunoprecipitation; K24, lysine 24; MARCH, Membrane-Associated RING-CH.

IFITM3 has four lysine residues: K24, K83, K88, and K104, which can serve as ubiquitination sites. To determine which of these four lysines are subject to ubiquitination by MARCH8, we replaced each lysine with an arginine (Fig. 4*A*). Coexpression analysis showed that MARCH8 downregulated the protein levels of IFITM3 K83R and K104R mutants, whereas the K88R mutant exhibited a nonsignificant reduction trend. Notably, both the K24R single mutant and 4KR mutant maintained stable expression profiles unaffected by MARCH8 (Figs. 4, *B* and *C* and S7, *A* and *B*). Interestingly, the 4KR mutant maintained colocalization with MARCH8 (Fig. 4*D*). Consistently, MARCH8 did not increase ubiquitination of the K24R and 4KR mutants (Fig. 4*E*). In addition, we engineered mutants with a single lysine retained and the other lysines replaced by alanine. The results showed that K88 was also partly ubiquitinated in addition to K24 (Fig. S8). These data collectively support Lys24 of IFITM3 as the primary site of MARCH8-mediated ubiquitination.

It has been shown that deletion of the first 21 amino acids or the YLAA mutation causes IFITM3 to localize on the plasma membrane and dampens IFITM3 antiviral function (49). To investigate the effect of MARCH8 on these IFITM3 mutants, we constructed these two mutants and found that MARCH8 did not downregulate the expression of D1-21 or the YLAA mutant, which altered the localization of IFITM3 and affected its antiviral function (Fig. 4, *F* and *G*). Surprisingly, MARCH8 still induced ubiquitination of these two mutants (Fig. 4*H*). These results indicate that the IFITM3’s endocytic motif is also pivotal in the MARCH8-mediated degradation of IFITM3.

### MARCH8 regulates IFITM3 turnover, localization, and trafficking

**Figure 5:**
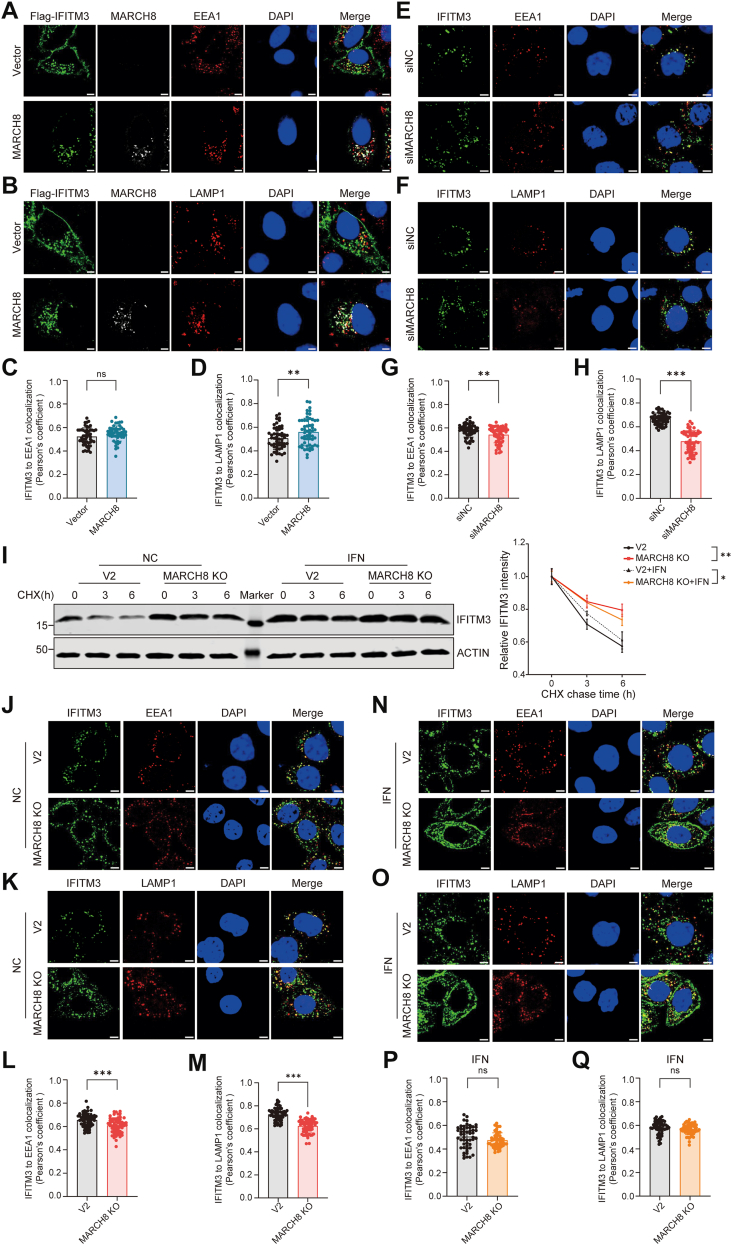
**MARCH8 regulates IFITM3 turnover, localization, and trafficking.***A*–*D,* HeLa cells were transfected with FLAG-IFITM3 and vector or EGFP-MARCH8, then permeabilized, costained with FLAG and EEA1 antibodies (*A*) or LAMP1 antibodies (*B*). Statistical result of the colocalization analysis is shown on the figures (*C* and *D*). Scale bars indicate 5 μm in all panels. Values are means ± SD from >50 cells (n = 3 independent experiments). Statistical differences were determined by two-sided Student’s *t* test; ∗∗*p* < 0.001, ns, nonsignificant. *E*–*H,* HeLa cells were transfected with siRNA targeting MARCH8 or control siRNA, then costained with IFITM3 and EEA1 antibodies (*E*) or LAMP1 antibodies (*F*). Statistical result of the colocalization analysis is shown on the figures (*G* and *H*). Scale bars indicate 5 μm in all panels. Values are means ± SD from >50 cells (n = 3 independent experiments). Statistical differences were determined by two-sided Student’s *t* test; ∗∗∗*p* < 0.0001, ∗∗*p* < 0.001. *I,*MARCH8 WT and KO HeLa cells were treated by IFN-α (250 μM) or DMSO. After 24 h, the cells were treated by CHX (100 μM) for 0, 3, and 6 h followed by Western blot. The quantification is shown on the *right* and derives from three independent experiments (mean ± SD; unpaired *t* test). Statistical differences were determined by two-sided Student’s *t* test; ∗∗*p* < 0.001, ∗*p* < 0.01. *J*–*Q,* MARCH8 WT and KO HeLa cells were treated by DMSO (*J*, *K*) or IFN-α (*N*, *O*), then costained with IFITM3 and EEA1 antibodies (*J*, *N*) or LAMP1 antibodies (*K*, *O*). Statistical result of the colocalization analysis is shown on the figures (*L*, *M* and *P*, *Q*). Scale bars indicate 5 μm in all panels. Values are means ± SD from >50 cells (n = 3 independent experiments). Statistical differences were determined by two-sided Student’s *t* test; ∗∗∗*p* < 0.0001, ns, nonsignificant. CHX, cycloheximide; DMSO, dimethyl sulfoxide; EGFP, enhanced GFP; HEK293T, human embryonic kidney 293T cell line; IFITM3, interferon-induced transmembrane protein 3; IFN-α, interferon alpha; MARCH, Membrane-Associated RING-CH.

The intracellular localization of IFITM3 plays an important role in its antiviral activity and other physiological functions (49). Next, we investigated whether MARCH8 may change the distribution of IFITM3 between endosomes and lysosomes by immunofluorescence microscopy. Using endosome marker EEA1 and lysosome marker LAMP1, we found that MARCH8 redistributed IFITM3 from the plasma membrane to lysosomes (Fig. 5, *A*–*D*). Knockdown of MARCH8 decreased the localization of IFITM3 to endosomes and lysosomes (Fig. 5, *E*–*H*). These data suggest that MARCH8 supports the lysosomal degradation and turnover of IFITM3.

To investigate the effect of MARCH8 on IFITM3 under physiological or pathological conditions, we examined protein dynamics following IFNα treatment, which mimics endogenous IFITM3 induction patterns observed *in vivo*. IFN treatment significantly upregulated IFITM3 protein levels. Strikingly, MARCH8-KO cell lines exhibited substantially delayed IFITM3 degradation kinetics compared with control, regardless of IFN treatment status (Fig. 5*I*). Our experimental data support the hypothesis that MARCH8-mediated post-translational regulation may serve as a key regulatory mechanism contributing to IFITM3 protein homeostasis maintenance under physiological conditions. We further investigated the role of MARCH8 in regulating the intracellular distribution of IFITM3 following IFN stimulation. The localization of IFITM3 in the MARCH8-KO cell line was consistent with that in the MARCH8-knockdown cells (Fig. 5, *J*–*M*). The localization of IFITM3 to endosomes and lysosomes was not statistically distinguishable between MARCH8-KO cells and controls following IFN treatment (Fig. 5, *N*–*Q*). Interestingly, we found that IFITM3 protein was significantly enriched at the plasma membrane in IFN-stimulated MARCH8-KO cells compared with the control cells (Fig. S9). In conclusion, our findings indicate that MARCH8 regulates the intracellular trafficking of IFITM3 from the plasma membrane to endosomes and lysosomes for subsequent degradation.

### MARCH8 regulates cellular susceptibility to virus infection by controlling IFITM3 levels

**Figure 6:**
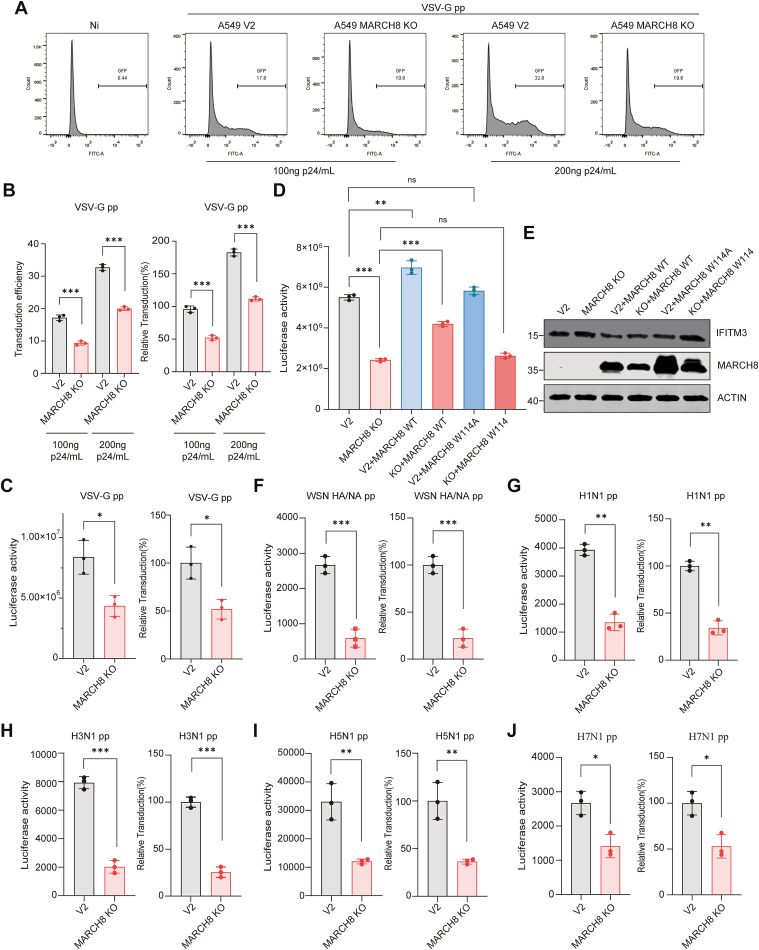
**MARCH8 KO increases IFITM3 level and protects cells from virus entry**. *A*–*C,* MARCH8 WT and KO A549 cells were transduced with 100 or 200 ng p24/ml VSV G pseudoviral particles (VSV G pp) for 48 h. Half of the cells were then fixed and examined for GFP positivity to measure the percentage of cells infected using flow cytometry (*A* and *B*), the other half of the cells were lysed to measure luciferase activity (*C*). Values are means ± SD from three independent experiments (n = 3). Statistical differences were determined by two-sided Student’s *t* test; ∗∗∗*p* < 0.0001. *D* and *E,* MARCH8 WT and KO HeLa cells were transfected with vector, MARCH8 WT, or W114A for 24 h and then transduced with VSV G pp for 48 h. The cells were lysed; luciferase activity was measured to report viral infection (*D*). MARCH8 levels and the effect of MARCH8 WT and W114A on endogenous IFITM3 levels were analyzed by Western blot (*E*). Values are means ± SD from three independent experiments (n = 3). Statistical differences were determined by two-sided Student’s *t* test; ∗∗∗*p* < 0.0001, ∗∗*p* < 0.001. ns, nonsignificant. *F*–*J,* MARCH8 WT and KO A549 cells were incubated with HIV-luc pseudoviral virus particles with the indicated viral envelope proteins. HA proteins from various influenza A virus strain include H1 (PR): A/PR/8/34 (H1N1), H3 (Udorn): A/Udorn/72 (H3N2), H5 (Thai): A/Thailand2 (SP-33)/2004 (H5N1), H7 (FPV): A/FPV/Rostock/34 (H7N1). Luciferase activity was measured to report viral infection. Values are means ± SD from three independent experiments (n = 3). Statistical differences were determined by two-sided Student’s *t* test; ∗∗∗*p* < 0.0001, ∗∗*p* < 0.001, and ∗*p* < 0.01. IFITM3, interferon-induced transmembrane protein 3; MARCH, Membrane-Associated RING-CH; VSV G, vesicular stomatitis virus glycoprotein.

IFITM3 inhibits many medically important viruses, such as HIV, VSV, severe acute respiratory syndrome coronavirus, and EBOV, and is active against many strains of influenza virus that have been tested to date, regardless of serotypes or species of origin (36, 38, 39, 41, 50, 51). Given the negative impact of MARCH8 on the level of IFITM3, we suspected that MARCH8 KO may render cells more resistant to virus infection. Indeed, a much lower number of MARCH8-KO cells were infected with VSV GP pseudotyped viruses expressing both GFP and luciferase as reporter than the control cells (Figs. 6, *A*–*C* and S10*A*). To determine whether elevated IFITM3 levels underline enhanced viral resistance in MARCH8 KO cells, we knocked down IFITM3 in both MARCH8 WT and KO cell lines 24 h before transduction. IFITM3 knockdown significantly increased viral susceptibility in both cell lines and eliminated the enhanced resistance phenotype in MARCH8-KO cells (Fig. S10, *B* and *C*). We next assessed the effect of MARCH8 coexpression with either IFITM3 WT or the ubiquitination-deficient IFITM3 4KR mutant. Following viral transduction, coexpression of MARCH8 with IFITM3 WT reduced IFITM3 protein levels, correlating with enhanced viral infection efficiency (Fig. S10, *D* and *E*). In contrast, coexpression of MARCH8 with the 4KR mutant had no significant effect on either protein abundance or transduction efficiency (Fig. S10, *D* and *E*). These results indicate that MARCH8 modulates viral infection primarily through ubiquitination-dependent regulation of IFITM3 stability. Furthermore, reexpression of WT MARCH8, but not the catalytically inactive MARCH8 W114A mutant, in MARCH8-KO cells resulted in a significant increase in VSV-G pseudovirus infection (Fig. 6*D*). Results of Western blotting showed that the supplement of the MARCH8 WT decreased IFITM3 expression, whereas the W114A mutant exerted no effect (Fig. 6*E*). We further showed that MARCH8-KO cells resisted infection of different influenza virus strains, including WSN, PR8, H1N1, H3N1, H5N1, and H7N1 (Fig. 6, *F*–*H*). These findings collectively demonstrate that MARCH8 promotes cellular susceptibility to viral infection through its regulatory role in suppressing IFITM3 protein levels.

## Discussion

As a membrane E3 Ub ligase, MARCH8 has been reported to ubiquitinate a battery of both cellular and viral proteins, which alters the property of these target proteins, including their stability, subcellular localization, and interactomes. In the context of viral infection, the functional consequences can vary, which is well reflected in both antiviral and proviral activities of MARCH8. For example, MARCH8 ubiquitinates the Env GPs of HIV-1, VSV, and EBOV, leading to protein degradation and consequent decrease of viral Env protein in virus particles (15, 16, 17, 18, 19, 20, 21, 22, 23). In the meantime, ubiquitination of NS2 protein of HCV by MARCH8 allows the recruitment of endosomal sorting complexes required for transport complex, which promotes HCV envelopment, and this proviral activity of MARCH8 has also been observed for Zika and dengue viruses (27, 28). MARCH8 can also facilitate viral replication by targeting cellular proteins. One example is the downregulation of the CUL1–UBE2L3 complex by MARCH8, which saves the E7 protein of HPV16 from being targeted for degradation (30). In this study, we discovered that MARCH8 ubiquitinates an IFN-stimulated gene product IFITM3, this provides a mechanism of regulating IFITM3 expression and potentially renders cells more susceptible to viral infection.

MARCH8 is not the only E3 Ub ligase that modifies IFITM3. NEDD4 has been previously reported to bind to the PpxY motif at the N-terminal region of IFITM3 and ubiquitinates it, thereby accelerating IFITM3 degradation (48). Consequently, NEDD4 knockdown or KO stabilizes IFITM3 protein levels, which increases cellular resistance to influenza virus infection (48). Our data showed that MARCH8 exhibits similar effects by the ubiquitination of K24 in IFITM3. One key difference is that MARCH8-mediated IFITM3 ubiquitination is independent of the PpxY motif. In addition, MARCH8 ubiquitinates IFITM3 with K63 Ub linkage, whereas NEDD4 modifies IFITM3 primarily with K48 Ub linkage. It appears that IFITM3 is subject to the ubiquitination and regulation by multiple cellular E3 Ub ligases, which of these E3 ligases may play a more dominant role may depends on their expression profiles and the availability of cofactors.

The seemingly opposing roles of MARCH8 in virus infections might be context dependent. For a specific virus, such as HPV16, MARCH8 may only act as a proviral factor and does not inhibit HPV16 at any stage of virus replication (30). For viruses, such as influenza virus, both antiviral and proviral activities have now been reported for MARCH8. For example, MARCH8 inhibits influenza virus by degrading viral protein M2 through ubiquitination at the late stage of viral replication (24). Using HA/NA pseudotyped virus, we showed in this study that MARCH8 increases HA-mediated virus entry by degrading IFITM3, which otherwise restricts the entry of influenza virus. The overall effect of MARCH8 on influenza virus infection might be a balance of its proviral and antiviral activities. Since knocking down MARCH8 in either cultured cells or mice promotes the infection of influenza virus, we envision that the antiviral effect of MARCH8 may outweigh its proviral effect.

As an IFN-stimulated gene, the main function of IFITM3 is to inhibit viral infections, which is well supported by the wide range of viruses under IFITM3 restriction, vulnerability of IFITM3 KO mice to lethality with influenza virus infection, as well as the association of IFITM3 polymorphisms in humans with higher susceptibility to viral diseases (37, 52, 53, 54, 55). In addition, IFITM3 is also involved in several important cellular processes. For example, excessive IFITM3 levels have been associated with pregnancy complications during congenital infections and IFN-induced pathologies, because IFITM3 has the ability of hindering syncytiotrophoblast formation and syncytin-mediated fusion (44). Recent studies have shown that inflammatory factors, which induce IFITM3 expression in neurons and astrocytes, can elevate its levels when combined with increased gamma-secretase activity. This, in turn, boosts beta-amyloid production, increasing the risk of Alzheimer’s disease. Notably, IFITM3 expression rises with age and is observed in mouse models harboring familial Alzheimer’s disease genes, suggesting a link of IFITM3 levels to Alzheimer’s disease susceptibility (43). Elevated IFITM3 expression has been observed in various tumors, with its levels correlating with histopathological grade and stage. Meanwhile, IFITM3 knockdown also inhibits migration, invasion, and proliferation of tumor cells (42, 56). In the context of B –cell development and activation, IFITM3 amplifies B-cell receptor signaling by acting as a scaffold *via* binding to PIP3 and orchestrating the assembly of B-cell receptor complex (42). These findings highlight the importance of regulating IFITM3 expression and cellular localization to warrant its normal physiological functions. In this regard, abnormal expression of IFITM3 may lead to diseases. It is possible that MARCH8 may contribute to establishing the optimal expression of IFITM3 in various tissues and under different conditions.

In summary, we have identified a previously unknown activity of MARCH8 in ubiquitinating and downregulating the antiviral protein IFITM3, thus may provide a negative feedback mechanism to prevent excessive immune activation. This finding adds to our understanding of the regulatory schemes governing host restriction factors in the context of innate immune response against viral infections. Given that high IFITM3 levels have been reported in a range of physiological dysregulations and pathological conditions, the ability of MARCH8 in downregulating IFITM3 expression may offer potential strategies for the development of effective medical interventions.

## Experimental procedures

### Cell culture

HEK293T cells (CRL-2316), human lung adenocarcinoma cells (A549, CCL-185), and human cervical cancer cells (HeLa, CCL-2) were purchased from the American Type Culture Collection. All cells were cultured in Dulbecco’s modified Eagle’s medium with 10% fetal bovine serum (Hyclone), 1% penicillin (100 U/ml), and streptomycin (100 μg/ml) (Thermo Fisher Scientific). All cells were cultured at 37 °C in a humidified incubator with 5% CO_2_ and certified negative for mycoplasma.

### Plasmids and reagents

Human MARCH8-expressing plasmids pQCXIP-MARCH8 and its RING-CH domain mutant pQCXIP-MARCH8-W114A were constructed by inserting the MARCH8 complementary DNA into the NotI–BamHI sites in the pQCXIP retroviral vector (Clontech). Human IFITM3-expressing plasmids pQCXIP-IFITM3 and its K/R mutant were constructed by plasmid loop expansion. Antibodies used for Western blot are as follows: MARCH8 (Proteintech; catalog no.: 14119-1-AP; 1:1000 dilution), IFITM3 (Proteintech; catalog no.: 11714-1-AP; 1:1000dilution), FLAG (Sigma–Aldrich; catalog no.: F7425; 1:2000 dilution), Myc (Sigma–Aldrich; catalog no.: C3956; 1:2000 dilution), HA.11 Epitope Tag (BioLegend; catalog no.: 901501 or catalog no.: 902301; 1:1000 dilution), and actin (Sigma–Aldrich; catalog no.: A1978; 1:2000 dilution).

### Western blotting analysis

Cells were lysed with radioimmunoprecipitation assay lysis buffer containing protease inhibitor cocktail. Lysates were centrifuged at 12,000*g* for 10 min, supernatants were collected, protein concentration was measured by a bicinchoninic acid assay. After incubation with SDS loading buffer at 95 °C for 10 min, the protein samples were resolved by 10% SDS-PAGE, transferred to polyvinylidene difluoride membranes, and blocked with Tris-buffered saline containing 5% (w/v) bovine serum albumin before being incubated with primary antibodies at room temperature for 2 h or at 4 °C overnight, and with secondary antibodies for 1 h at room temperature. The Western blot images were acquired and analyzed with LICOR Odyssey CLx with ImageStudio lite v5.2 software. Densitometric analysis was performed with ImageJ (National Institutes of Health, NIH) software.

### Immunoprecipitations

Cells were lysed in IP lysis buffer (50 mM Tris–HCl [pH 7.5], 150 mM NaCl, 0.2% Triton X-100, and 10% glycerol) supplemented with protease inhibitor cocktail. The debris was removed by centrifugation at 12,000*g* for 10 min. A portion of the supernatant (80 μl) was retained as the whole-cell lysate, and the rest was incubated with agarose beads conjugated with different antibodies at 4 °C for 2 h. IP materials were washed three times with IP lysis buffer. Proteins were eluted from the beads by boiling in SDS loading buffer and then analyzed by Western blotting.

### Mass spectrometry

HeLa cells were transfected with FLAG-MARCH8 plasmid or empty vector. Cells were then treated with lysosomal inhibitor CQ and lysed in IP lysis buffer and centrifugation at 12,000*g* for 10 min. A portion of the supernatant was incubated with anti-FLAG beads at 4 °C for 2 h. The beads were washed three times with IP lysis buffer and then were excised from the beads for MS identifications (Beijing Biotech-Pack Scientific). The raw MS files were analyzed and searched against protein database based on the species of the samples using MaxQuant (the Max Planck Institute of Biochemistry). Only proteins with at least two unique peptides were analyzed. The proteins identified in the negative controls were subsequently excluded from the list of MARCH8-interacting proteins, and then the specifically bound proteins were subjected to subsequent bioinformatics analyses.

### Immunofluorescence microscopy

IF was performed with cells 24 h post transfection with IFITM3 and MARCH8 plasmid DNA. Early or late endosomes were labeled by anti-EEA1 (BD Biosciences; catalog no.: 610456; 1:250 dilution) or anti-LAMP-1 (BioLegend; catalog no.: 328602; 1:250 dilution) antibody (Ab). The cell membrane was labeled by Alexa Fluor 555–conjugated CtxB (Invitrogen Life Technologies; catalog no.: C34776; 1:500 dilution). Confocal images were collected with Leica TCS SP8 confocal microscope and analyzed by LAS AF Lite v3.0 and ImageJ v1.44.

### BiFC assay

The direct binding of MARCH8 and IFITM3 was verified by using BiFC as previously described (45). Briefly, DNAs from human MARCH8, NEDD4, and IFITM3 were inserted into BiFC vectors pBiFC-HA-VC155 and pBiFC-FLAG-VN173, respectively. The HeLa cells were cotransfected with the recombinant plasmids. After 24 h, cells were costained with FLAG (Sigma–Aldrich; catalog no.: F7425; 1:1000 dilution) and HA (BioLegend; catalog no.: 901501; 1:1000 dilution) antibodies, and the confocal images were collected with Leica TCS SP8 and analyzed by LAS AF Lite v3.0.

### RNA interference

Gene silencing was performed using sequence-specific siRNAs. For HeLa cell experiments, three MARCH8- or IFITM3-targeting siRNAs (Sigma–Aldrich) were pooled at equimolar concentrations, with a nontargeting siRNA serving as a negative control. Transfection was conducted using Lipofectamine RNAiMax transfection reagent (Invitrogen; catalog no.: 13778150) according to the manufacturer’s instructions. After 24 h of incubation, knockdown efficiency was confirmed by RT–quantitative PCR (qPCR) and Western blot analysis. Target-specific siRNA sequences are provided in Table S1.

### Generation of MARCH8-KO cells

Generation of MARCH8-KO cells was performed as previously described (24). To generate cell lines that stably express MARCH8, cells were transduced with pQCXIP (Clontech)-based pseudovirus according to the manufacturer’s instruction. To generate MARCH8-KO cell lines, a lentiviral CRISPR–Cas9 expression plasmid pCRISPR-MARCH8 was created by inserting DNA fragments that contain a target sequence of MARCH8 (1#: 5′-GTAAGACCAAAGAAAAGGAG-3′ or 2#: 5′-GAGCTCGCAGCAGCGCGTGT-3′) into lentiCRISPR-V2 (Addgene). Cells were transduced with pseudoviruses expressing control CRISPR–Cas9 (V2) or CRISPR–Cas9 targeting MARCH8. Stably transduced cell lines were selected with puromycin, and cell clones were generated with limited dilution. Single clonal cell lines were screened *via* Western blot.

### Real-time qPCR analysis

Total cellular RNA was extracted using the PureLink RNA Extraction kit (Thermo Fisher Scientific) according to the manufacturer’s protocol. Complementary DNA was synthesized using the PrimeScript RT reagent Kit with gDNA Eraser (TaKaRa). An oligo(dT)20 primer was used for RT of cellular mRNA. B-actin mRNA was used as an internal control. The sequences of primers used in qPCRs are provided in Table S2. Real-time qPCR assays were further analyzed by Bio-Rad CFX Manager.

### Flow cytometry

Flow cytometry analysis was performed as previously described (24). Briefly, cells were detached and resuspended in complete growth media. Cells were fixed with 4% paraformaldehyde for 10 min at room temperature. Cells were then incubated for 1 h with an Ab (1:100 dilution), followed by staining for 1 h with a rabbit anti-mouse IgG conjugated with Alexa Fluor 488 or 555 (Thermo Fisher; A21446, A21446; 1:1000 dilution). Cells were analyzed using BD FACSCanto II Flow Cytometer. The data were collected and analyzed with BD FACSDiva Software v8.0 software and Flowjo v10.

### Detection of ubiquitination by IP

Cells were treated with CQ (50 μM) for 4 h and lysed in IP lysis buffer containing DUB inhibitors (100 mM PR619, 5 mM 1,10-phenanthroline, and 5 mM *N*-ethylmaleimide), and a protease inhibitor cocktail. Lysates spun at 12,000*g* for 10 min. A portion of the supernatant was retained as the whole-cell lysate, and the rest was incubated with Ab-coated agarose beads at 4 °C for 2 h. The beads were then added to the clarified supernatants for 2 h at 4 °C. Beads were washed with catch and release IP wash buffer (Invitrogen) and eluted in 2x SDS sample buffer. The samples were then analyzed by Western blotting.

### Pseudovirus production and transduction

VSV pseudoviruses were produced by transfecting HEK293T cells with 9 μg of the packaging plasmid psPAX2, 3 μg of the reporter plasmid Lenti-GFP/Luciferase, and 3 μg of the VSV-G Env protein plasmid. Influenza pseudoviruses were generated using the same method, with the Env protein plasmid replaced by 3 μg pCAGGS-HA and 3 μg pCAGGS-NA. Forty-eight hours after transfection, the supernatant was collected and passed through a 0.2 μm filter. Viral particle production was quantified by p24^Gag^ ELISA. To determine transduction efficiency, equal amounts of p24^Gag^ antigen of pseudoviruses were used to infect cells in 12-well plates at a density of 2 × 10^5^ per well. Influenza pseudoviruses were conducted in virus maintenance medium (Dulbecco’s modified Eagle’s medium supplemented with 0.5% fetal bovine serum, 1 μg/ml PTCK–trypsin, and a 1% penicillin–streptomycin mixture). At 48 h after transduction, cells were lysed with 1× cell lysis buffer (Promega; E1531), and the luciferase activities were determined using the Luciferase assay system (Promega; E1500).

### Luciferase activity assay

Cells transduced with a luciferase reporter pseudovirus were collected and lysed in luciferase lysis buffer, then centrifugated at 12,000*g* for 10 min. The supernatant was supplemented with luciferase substrate, after which luciferase activity was measured according to the manufacturer’s protocol.

## Statistical analysis

Statistical analysis was performed with GraphPad Prism 8.0 software (GraphPad). Unless otherwise indicated, graphs display mean ± SD and represent data from at least three independent experiments. Statistical significance was analyzed using two-tailed unpaired Student’s *t* test. Significance indicated by asterisks is designated as follows: ∗*p* < 0.01; ∗∗*p* < 0.001; ∗∗∗*p* < 0.0001; and ns, nonsignificant.

## Statistics and reproducibility

For *in vitro* experiments, phenotypic analyses including immunofluorescence, Western blot, and fluorescence-activated cell sorting were performed in least three independent experiments, using biological replicates.

## Data availability

The MS proteomics data have been deposited to the ProteomeXchange Consortium (https://proteomecentral.proteomexchange.org) *via* the iProX partner repository (57, 58) with the dataset identifier PXD062157. The data that support the findings of this study are available on request from the corresponding author.

## Supporting information

This article contains supporting information

## Conflict of interest

The authors declare that they have no conflicts of interest with the contents of this article.
