# Supplementary material for: MARCH8-mediated ubiquitination regulates expression of the antiviral protein IFITM3
Source: J Biol Chem. 2025 Nov 4;301(12):110879. doi: 10.1016/j.jbc.2025.110879 (PMC12702058; doi:10.1016/j.jbc.2025.110879)
Supplement: Supplementary Figures [file mmc1.docx]

**Supplementary Figures**

**MARCH8-mediated ubiquitination regulates expression of the antiviral protein IFITM3**

**Liang Wei^1, 2 †^, Fei Zhao^1, 2 †^, Xiaoman Liu^1, 2 †^, Shan Mei^1, 2^, Yu Huang^1, 2^, Yu Xie^1, 2^, Yamei Hu^1, 2^, Liming Wang^1, 2^, Lingwa Wang^3^, Zhao Gao^1, 2^, Chen Chen^1, 2^, Yueyue Shi^3^, Yurong He^3^, Jiaxun Wang^1, 2^,** **Tiffany Xue^4^, Fengwen Xu^1, 2*^, Jugao Fang^3*^, Fei Guo^1, 2*^**

**Affiliations:**

^1^ Key Laboratory of Pathogen Infection Prevention and Control (Ministry of Education), State Key Laboratory of Respiratory Health and Multimorbidity

^2^ NHC Key Laboratory of Systems Biology of Pathogens, National Institute of Pathogen Biology and Center for AIDS Research, Chinese Academy of Medical Sciences & Peking Union Medical College, Beijing 100730, P. R. China

^3^ Department of Otolaryngology Head and Neck Surgery, Beijing Tongren Hospital, Capital Medical University, Beijing, 100730, P. R. China

^4^ Department of Chemistry, Bryn Mawr College, Bryn Mawr, Pennsylvania 19010, USA

^*^Corresponding author (Email: xanadufw@aliyun.com; [fangjugao@163.com](mailto:fangjugao@163.com)；guofei@ipb.pumc.edu.cn)


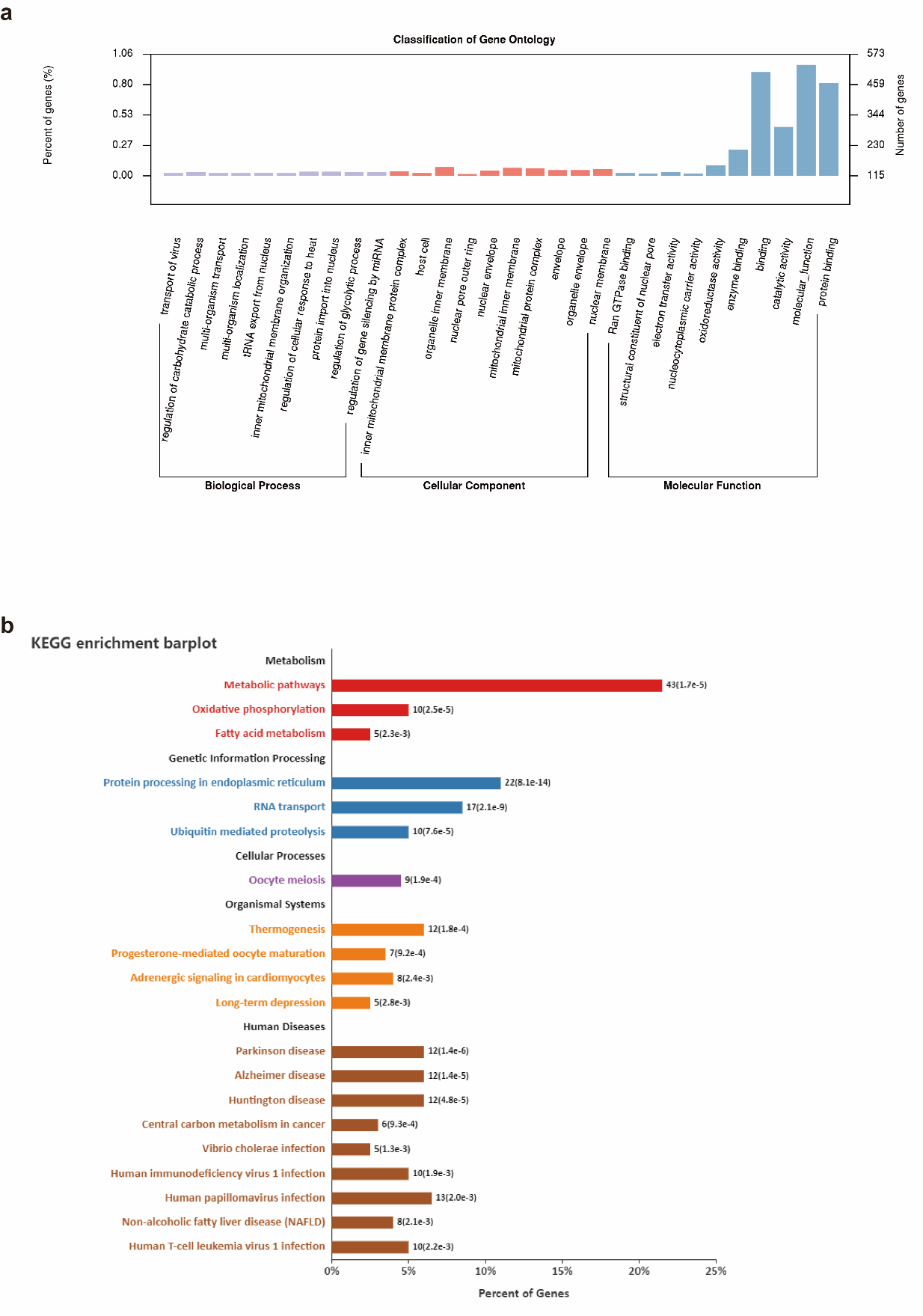


**Supplementary Figure 1. GO and KEGG pathway diagram of the differentially expressed proteins.**

a. Graphs representing GO analysis of the differentially expressed proteins.

b. KEGG pathway enrichment analysis map of differentially expressed genes.


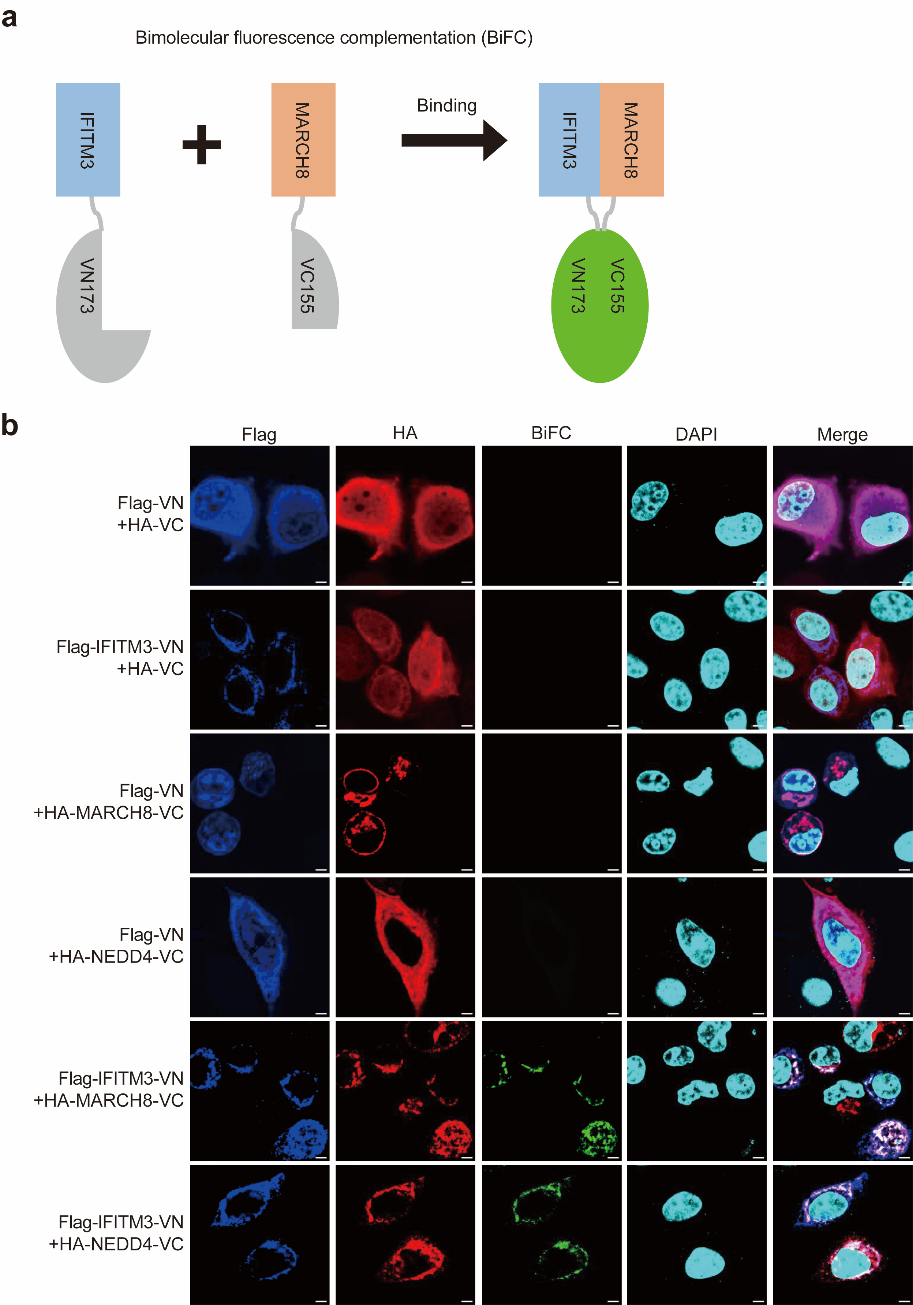


**Supplementary Figure 2. MARCH8 directly interacts with IFITM3.**

a. Scheme of MARCH8 and IFITM3 fusion proteins used for bimolecular fluorescence complementation (BiFC) analysis.

b. BiFC assay showing the interaction between IFITM3 with MARCH8 or NEDD4. HeLa cells were co-transfected with Flag-IFITM3-VN and either HA-MARCH8-VC- or HA-NEDD4-VC. Negative controls included cells transfected with Flag-VN and HA-VC. Cells were permeabilized and then co-stained with Flag and HA antibodies. Scale bars indicate 5 μm in all panels.


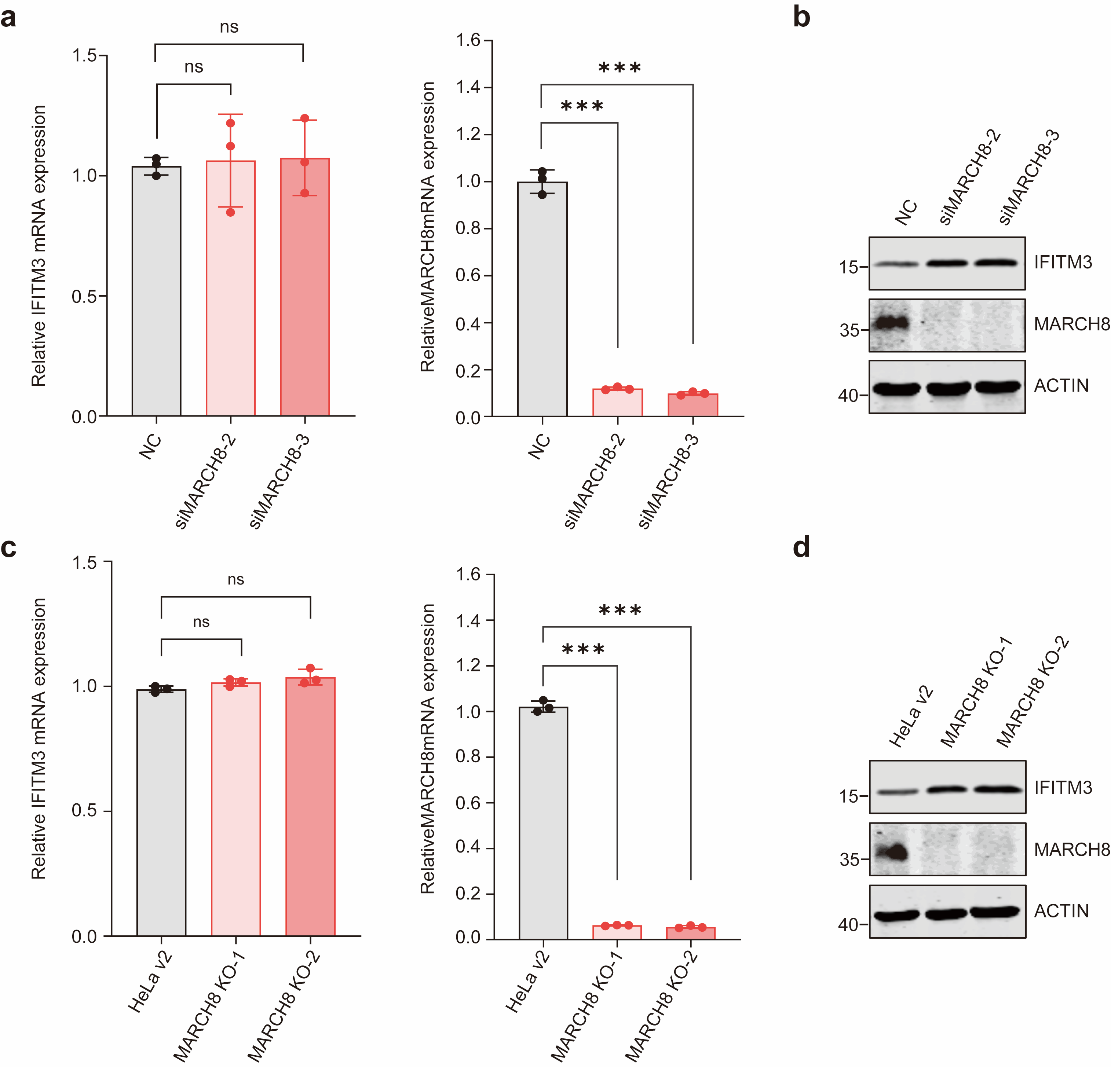


**Supplementary Figure 3. MARCH8 did not affect the transcription level of IFITM3.**

a. Effect of siRNA targeting MARCH8 on IFITM3 and MARCH8 RNA levels. Values were normalized to GAPDH. Values are means ± s.d. from three independent experiments (n = 3). Statistical differences were determined by two-sided Student’s t-test; ***P < 0.0001. n.s., nonsignificant.

b. The WB result of knockdown.

c. Relative RNA levels of IFITM3 and MARCH8 in HeLa MARCH8 KO cells compared to HeLa V2 control cells, as measured by RT-qPCR. Values were normalized to GAPDH. Values are means ± s.d. from three independent experiments (n = 3). Statistical differences were determined by two-sided Student’s t-test; ***P < 0.0001. n.s., nonsignificant.

d. The WB result of MARCH8 knockout.


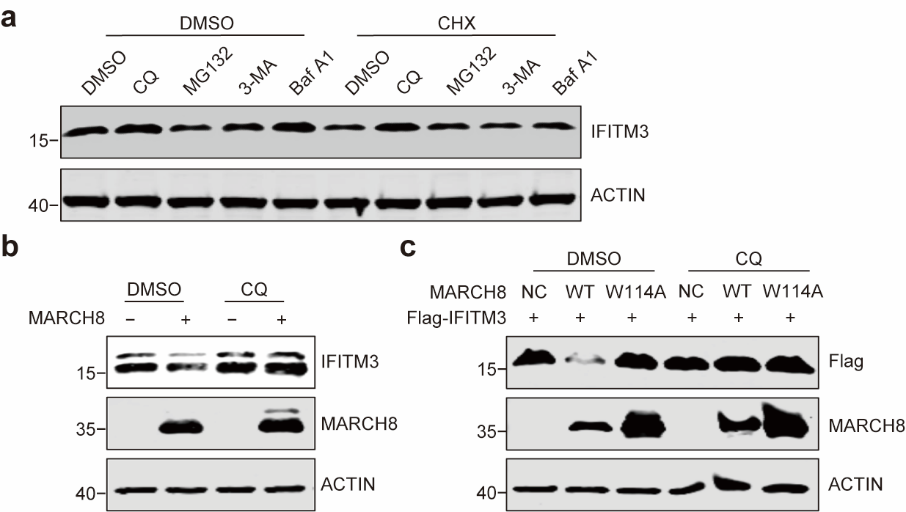


**Supplementary Figure 4. MARCH8 leads to IFITM3 degradation in lysosomes.**

a. Degradation of endogenous IFITM3 in response to inhibitors of different degradation pathways.

b. HeLa cells were transfected with MARCH8 WT or vector. Cells were treated with CQ (50 μM) for 4 h and processed for Western blot.

c. HEK293T cells were co-transfected with Flag-IFITM3 and MARCH8 WT, W114A or empty vector. Cells were then treated with DMSO and CQ (50 μM) for 4 h and processed for Western blot.


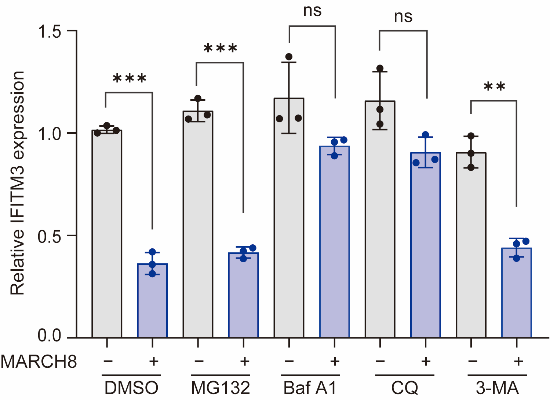


**Supplementary Figure 5. MARCH8 leads to IFITM3 degradation in lysosomes pathway.**

Statistical result of the Western blot (Fig 3C). Values are means ± s.d. from three independent experiments (n = 3). Statistical differences were determined by two-sided Student’s t-test; ***P < 0.0001. **P < 0.001. n.s., nonsignificant.


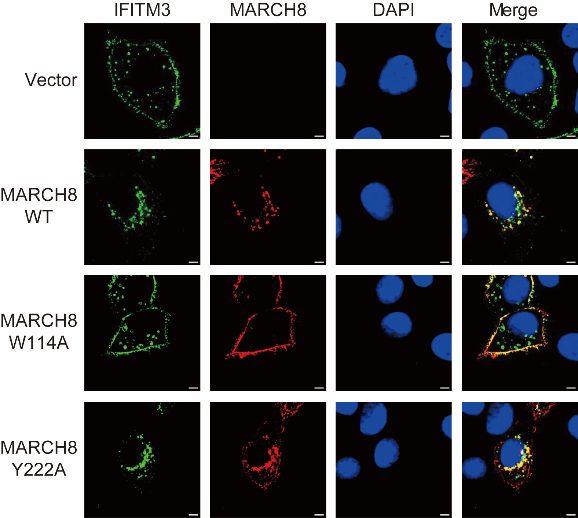


**Supplementary Figure 6. The colocalization of IFITM3 with MARCH8 protein mutants.**

HeLa cells were co-transfected with Flag-IFITM3 and MARCH8 WT, W114A, or Y222A. Cells were treated with CQ (50 μM) for 4 h and processed for immunofluorescence staining with anti-Flag and anti-MARCH8 antibodies.


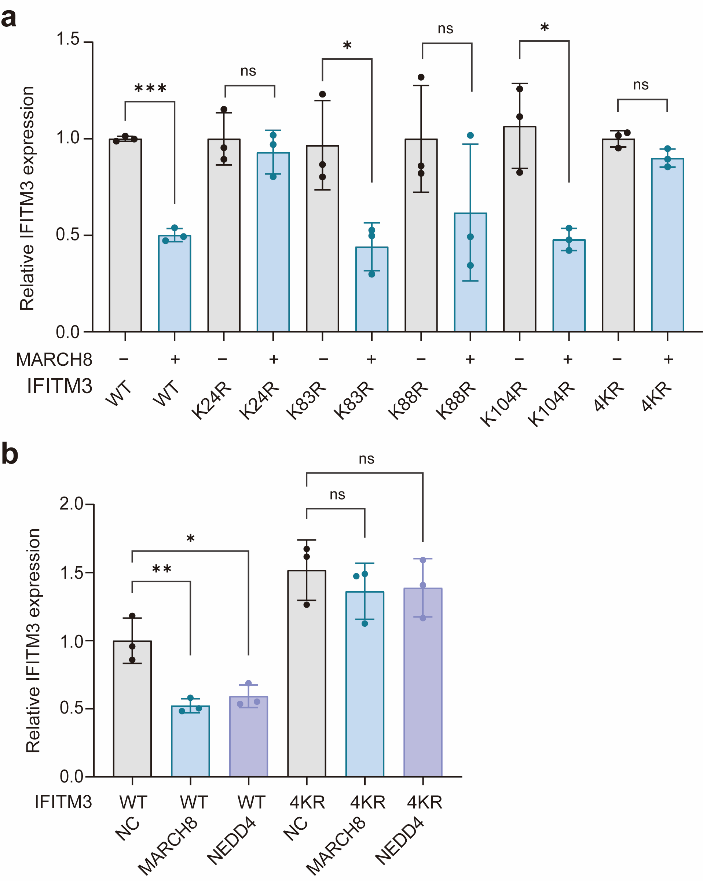


**Supplementary Figure 7. K24 is the primary ubiquitination site of IFITM3 by MARCH8.**

a. Statistical result of the Western blot (Fig 4B). Values are means ± s.d. from three independent experiments (n = 3). Statistical differences were determined by two-sided Student’s t-test; ***P < 0.0001. *P < 0.01. n.s., nonsignificant.

b. Statistical result of the Western blot (Fig 4C). Values are means ± s.d. from three independent experiments (n = 3). Statistical differences were determined by two-sided Student’s t-test; *P < 0.01. **P < 0.001. n.s., nonsignificant.


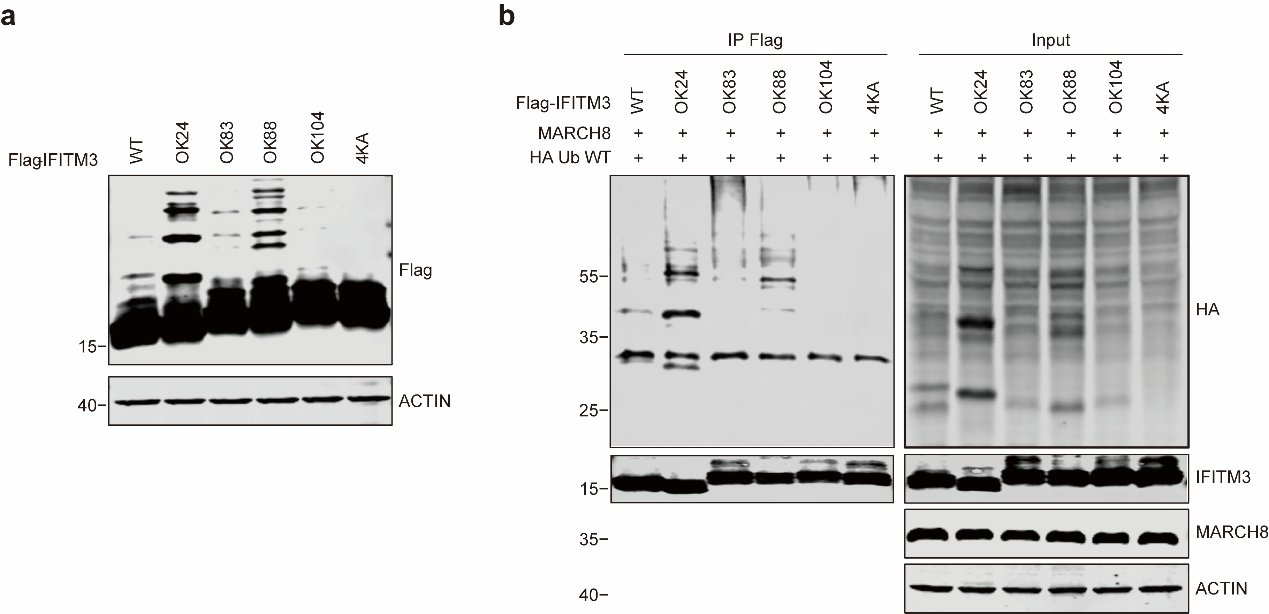


**Supplementary Figure 8. The ubiquitination of the IFITM3 mutants.**

a. HEK293T cells were transfected with Flag-IFITM3 WT and mutants that retained only the lysine residue (OK) followed by Western blot.

b. HEK293T cells were transfected with Flag-IFITM3 mutants (OK), HA-Ub WT and MARCH8. Cell lysates were subject to IP with anti-Flag antibody, and the IP and input samples were analyzed by Western blotting with antibodies against the indicated protein targets.


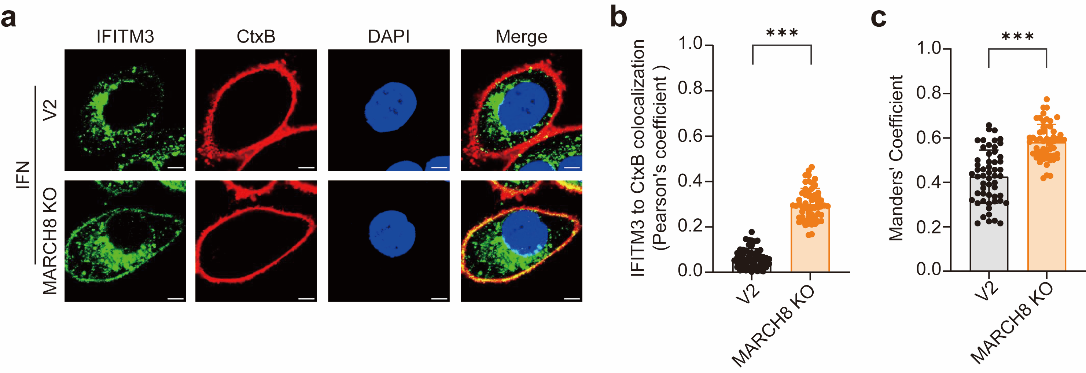


**Supplementary Figure 9. MARCH8 regulates IFITM3 localization, and trafficking.**

a. MARCH8 WT and knockout HeLa cells were treated by IFN-α, then co-stained with IFITM3 and cholera toxin B subunit (CtxB), which can interact with the lipid raft marker GM1 as a marker of cell membrane (a). Scale bars indicate 5 μm in all panels.

b and c. Statistical result of the colocalization analysis. Values are means ± s.d. from >50 cells cells (n = 3 independent experiments). Statistical differences were determined by two-sided Student’s t-test; ***P < 0.0001.


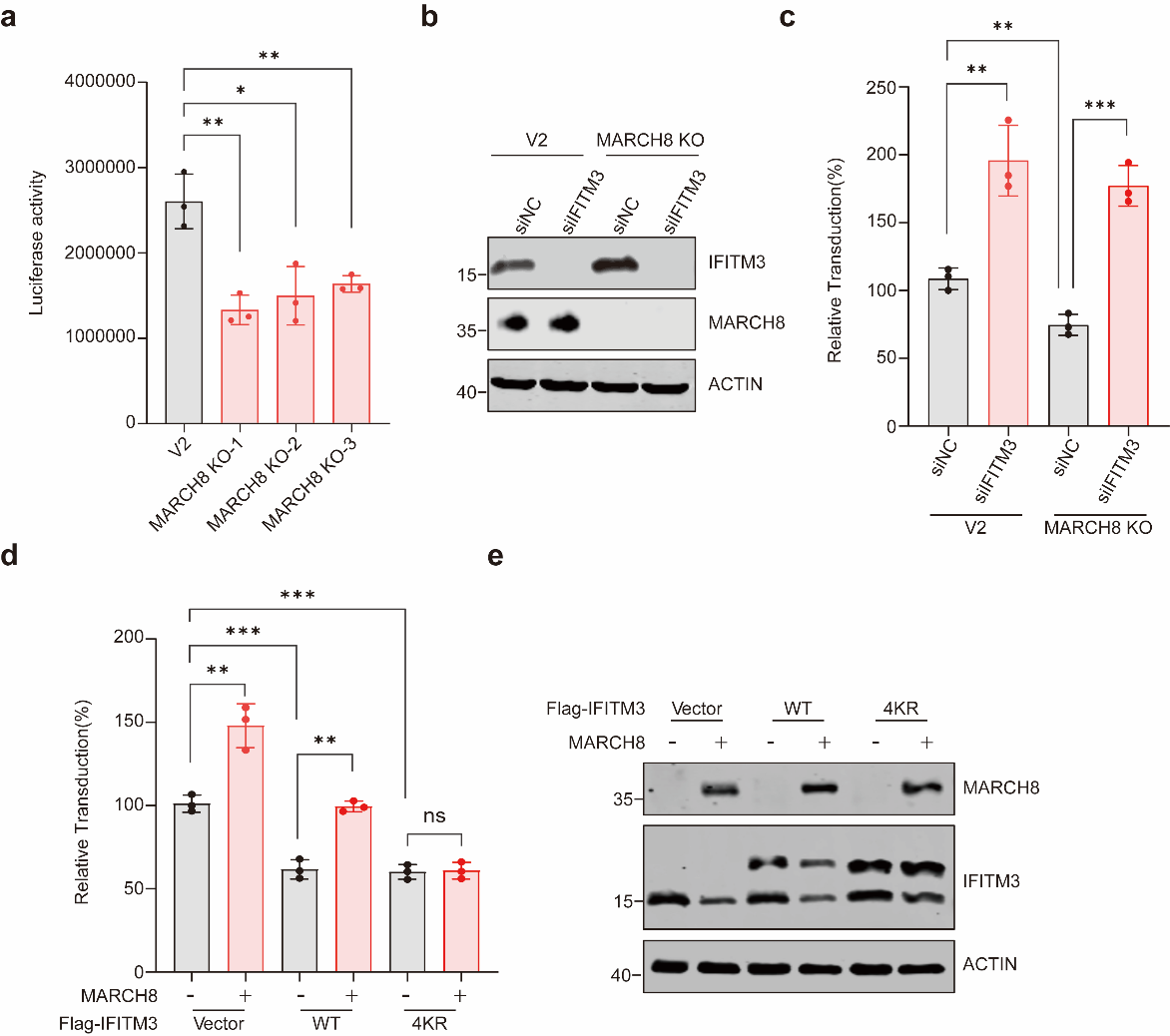


**Supplementary Figure 10. MARCH8 knockout cells protect cells from VSV entry.**

a. MARCH8 WT and knockout HeLa cells were transduced with VSV G pp for 48 h. Cells were lysed to measure luciferase activity, which was measured to report viral infection. Values are means ± s.d. from three independent experiments (n = 3). Statistical differences were determined by two-sided Student’s t-test; **P < 0.001. *P < 0.01.

b and c. MARCH8 WT and KO HeLa cells were transfected for 24 h with control siRNA (siNC) or siRNA targeting IFITM3 (siIFITM3). Cells were collected just prior to infection for confirmation of IFITM3 knockdown by anti-IFITM3 and MARCH8 Western blotting (b). Following siRNA treatment, cells were transduced with VSV G pp for 48 h. Cells were lysed to measure luciferase activity, which was measured to report viral infection (c). Values are means ± s.d. from three independent experiments (n = 3). Statistical differences were determined by two-sided Student’s t-test; ***P < 0.0001. **P < 0.001.

d and e. HeLa cells were transfected with vector, Flag-IFITM3 WT or 4KR and vector or MARCH8. After 24 h, cells were transduced with VSV G pp for 48 h. Cells were lysed to measure luciferase activity, which was measured to report viral infection (d). MARCH8 levels and the effect of MARCH8 on Flag-IFITM3 WT or 4KR levels were analyzed by Western blot (e). Values are means ± s.d. from three independent experiments (n = 3). Statistical differences were determined by two-sided Student’s t-test; ***P < 0.0001. **P < 0.001. n.s., nonsignificant.
