# Supplementary material for: MARCH8-mediated ubiquitination regulates expression of the antiviral protein IFITM3
Source: J Biol Chem. 2025 Nov 4;301(12):110879. doi: 10.1016/j.jbc.2025.110879 (PMC12702058; doi:10.1016/j.jbc.2025.110879)
Supplement: Supplementary Table 1 [file mmc2.docx]

Supplementary Table 1 The siRNA sequences.

| Gene | siRNA ID | Sense strand (5'-3') | Antisense strand (5'-3') |
| --- | --- | --- | --- |
| IFITM3-1 | SASI_Hs02_00354510 | CACACUGUCCAAACCUUCUdTdT | AGAAGGUUUGGACAGUGUGdTdT |
| IFITM3-2 | SASI_Hs01_00038803 | GAUCUUCCAGGCCUAUGGAdTdT | UCCAUAGGCCUGGAAGAUCdTdT |
| IFITM3-3 | SASI_Hs01_00038804 | CCACGUACUCCAACUUCCAdTdT | UGGAAGUUGGAGUACGUGGdTdT |
| MARCH8-2 | SASI_Hs01_00011068 | CUAUAAUAGAGUGAUCUAUdTdT | AUAGAUCACUCUAUUAUAGdTdT |
| MARCH8-3 | SASI_Hs01_00011070 | CCUUGUAUGUGCUCAUUGAdTdT | UCAAUGAGCACAUACAAGGdTdT |
