# Supplementary material for: MARCH8-mediated ubiquitination regulates expression of the antiviral protein IFITM3
Source: J Biol Chem. 2025 Nov 4;301(12):110879. doi: 10.1016/j.jbc.2025.110879 (PMC12702058; doi:10.1016/j.jbc.2025.110879)
Supplement: Supplementary Table 2 [file mmc3.docx]

Supplementary Table 2 Primers for qPCR

| Gene | Forward primer (5'-3') | Reverse primer (5'-3') |
| --- | --- | --- |
| MARCH8 | GGAAGAACAGAATGAGAAGA | GATGGCGTGATAGAAGTG |
| IFITM3 | GAATCACACTGTCCAAACCTTC | CATAGGCCTGGAAGATCAGC |
| β-Actin | ACCAACTGGGACGACATGGAGA  AA | TAGCACAGCCTGGATAGCAA  CGTA |
